# A norming study of high-quality video clips of pantomimes, emblems, and meaningless gestures

**DOI:** 10.3758/s13428-018-1159-8

**Published:** 2018-12-12

**Authors:** Beatrice Agostini, Liuba Papeo, Cristina-Ioana Galusca, Angelika Lingnau

**Affiliations:** 1 Center for Mind/Brain Science (CIMeC), University of Trento, 38068 Rovereto, TN Italy; 2 CNRS—Institut des Sciences Cognitives Marc Jeannerod—UMR 5304 and University Claude Bernard Lyon 1, Bron, France; 3 Center for Brain and Cognition, Universitat Pompeu Fabra, Ramon Trias Fargas, 25–27, 08005 Barcelona, Spain; 4 Department of Psychology, Royal Holloway University of London, Egham, London, TW20 0EX UK; 5 Department of Psychology and Cognitive Science, University of Trento, 38068 Rovereto, TN Italy; 6 Present Address: Institute of Psychology, University of Regensburg, 93053 Regensburg, Germany

**Keywords:** Gestures, Emblems, Pantomimes, Object-directed actions

## Abstract

Recent years have witnessed a growing interest in behavioral and neuroimaging studies on the processing of symbolic communicative gestures, such as pantomimes and emblems, but well-controlled stimuli have been scarce. This study describes a dataset of more than 200 video clips of an actress performing pantomimes (gestures that mimic object-directed/object-use actions; e.g., playing guitar), emblems (conventional gestures; e.g., thumbs up), and meaningless gestures. Gestures were divided into four lists. For each of these four lists, 50 Italian and 50 American raters judged the meaningfulness of the gestures and provided names and descriptions for them. The results of these rating and norming measures are reported separately for the Italian and American raters, offering the first normed set of meaningful and meaningless gestures for experimental studies. The stimuli are available for download via the Figshare database.

**Electronic supplementary material:**

The online version of this article (10.3758/s13428-018-1159-8) contains supplementary material, which is available to authorized users.

*Communicative symbolic gestures* are movements, most frequently performed with the upper limbs, that convey meaning even when they occur without speech. These gestures, including pantomimes of actions (e.g., *playing guitar*) and emblems (e.g., *waving goodbye*), are distinct from *co-speech gesticulations*, which acquire meaning only in the presence of speech (Ekman & Friesen, 1969; Kendon, 1988; McNeill, 2005). Pantomimes and emblems also differ from sign language, as they lack the combinatorial and linguistic complexity of a fully fledged language in the manual modality. They are present in most, if not all, cultures and play an important role in communication (McNeill, 2005; Goldin-Meadow, 1999, 2003; Xu, Gannon, Emmorey, Smith, & Braun, 2009).

To illustrate the difference across types of gestures, McNeill proposed a continuum (Kendon’s continuum) based on two dimensions, conventionality and co-occurrence with speech (adapted from McNeill, 2000), which placed symbolic gestures—pantomimes and emblems—right in between sign language and gesticulations. Though both pantomimes and emblems can convey meaning when speech is absent, these two types of gestures diverge greatly with respect to two critical features that determine their communicative value: iconicity and convention. *Iconic* gestures are characterized by a high correspondence between form and meaning (Ekman & Friesen, 1969). By contrast, gestures that are arbitrarily coded present little to no correspondence between form (i.e., the sign) and meaning. *Pantomimes* are the prototypical example of iconic gestures, in that they are faithful reproductions of common actions (Ekman, 2004; Poggi & Zomparelli, 1987); iconicity is the source of their meaning (McNeill, 1992; Perniss, Thompson, & Vigliocco, 2010; van Nispen, van de Sandt-Koenderman, & Krahmer, 2017). Instead, *emblems* are gestures that refer to meanings that are socially learned and transferred, and they have various degrees of iconicity: from a completely arbitrary form–meaning relationship (e.g., *thumbs up*, *waving goodbye*) to a moderate correspondence between form and meaning (e.g., *inviting someone to cut short* by repeatedly moving the index and middle fingers toward each other, to symbolically indicate scissors; Ekman, 2004; Ekman & Friesen, 1969; Gullberg, 2006; Molnar-Szakacs, Wu, Robles, & Iacoboni, 2007).

Kendon’s continuum, as opposed to a rigid categorization, emphasizes the transitory nature of distinctions between gestures. According to McNeill (2000), pantomimes are less conventional than emblems. That is, a novel pantomime produced spontaneously under no conventional constraints would be placed on the left side of the continuum. However, the same pantomime could be used repeatedly within a cultural group, subsuming the properties of emblems, as it becomes more precise, conventionalized, and abstracted away from its initial, purely iconic form (Brentari, Coppola, Mazzoni, & Goldin-Meadow, 2012; Sandler, Aronoff, Meir, & Padden, 2011). For example, the pantomime for “calling on a telephone” has become an emblem in many cultures due to its pervasive use in communication, with variations that reflect different conventions across cultures: Italians hold a fist with the thumb and little finger stretched out, as if connecting an ear to the mouth, whereas Americans hold a closed fist next to the cheek area, in between the mouth and the ear (Haviland, 2005).

Despite the pervasive use of gestures in communication to make requests, refer to actions or objects, represent mental states, and so on, it is largely unclear how individuals recognize and understand gestures. Most research has focused on co-speech gestures, showing how information from speech and gestures is integrated (Bernardis & Gentilucci, 2006; Kelly, Creigh, & Bartolotti, 2009; Kelly, Özyürek, & Maris, 2010; Özyürek, 2017; Özyürek, Willems, Kita, & Hagoort, 2007; Vainiger, Labruna, Ivry, & Lavidor, 2014; for a review, see Özyürek, 2014). Unlike co-speech gestures, symbolic gestures such as pantomimes and emblems are an ideal means to study how the brain assigns meaning, in the absence of linguistic cues (i.e., speech). A number of studies on pantomimes and emblems have addressed specific aspects of this question, such as the neural substrates of gesture recognition and understanding (Andric et al., 2013; Gunter & Bach, 2004; Kubiak & Króliczak, 2016; Papeo, Negri, Zadini, & Rumiati, 2010; Villareal et al., 2008; Xu et al., 2009), the cultural specificity of gestures (Kendon, 1995; Molnar-Szakacs et al., 2007), the relationship between the brain networks associated with language and symbolic gestures (Häberling, Corballis, & Corballis, 2016), as well as understanding of actions and gestures in brain-damaged patients (Kalénine, Buxbaum, & Coslett, 2010; Papeo & Rumiati, 2013; Tarhan, Watson, & Buxbaum, 2015).

This research has provided a rather heterogeneous (and not always consistent) picture of the neural and cognitive mechanisms involved in the processing of symbolic gestures, the relationship between the processing of pantomimes and of emblems, and the relationship between symbolic gestures and language (for a review, see Andric & Small, 2012). One reason for this heterogeneity is the high variability in the stimuli and tasks employed in various studies. Variability particularly occurs in the types of gestures selected for the study (emblems only, pantomimes only, or both) and in subtle but crucial aspects of the videos or the static images, such as the position of the actor (e.g., standing or sitting), the focus of the video/image (e.g., entire body, torso, or just hand), the background, and in the case of videos, the duration. Moreover, in different studies the stimuli have undergone different procedures for selection (e.g., different dimensions of the stimuli have been considered and rated in different studies). Crucially, not all previous studies on symbolic gestures have tested their comprehensibility and the overlap between the intended meaning (i.e., the meaning intended by the producer) and the meaning understood by the perceiver (i.e., the subject). All of these are sources of variability that could significantly affect the results of behavioral and neuroimaging studies and hinder replication of the observations from previous studies.

In the present study, we aimed to provide a publicly available database of high-resolution videos of pantomimes and emblems and a control set of meaningless gestures. Meaningless gestures are movements that do not evoke any conventional meaning that are designed to reproduce the surface features of the meaningful gestures and to provide stimuli for a control condition in behavioral and neuroimaging studies that seek to study the processing and representation of meaningful gestures. The gestures were based on the Italian culture and were performed by an Italian actress in Italy. To investigate possible cultural differences and evaluate the generalizability of the dataset to a non-Italian population, the gestures were rated by two groups of participants: Italians and Americans. Our aim was to provide a measure of the comprehensibility of these gestures across two cultures, by asking participants to complete three tasks: (1) judging the meaningfulness of each gesture, (2) naming the gesture (*naming task*), and (3) describing the meaning of the gesture (*description*). To the best of our knowledge, two sets of video clips are currently publicly available, one involving ballet dance movements (Christensen, Nadal, Cela-Conde, & Gomila, 2014) and another involving pantomimes (van Nispen et al., 2017). Therefore, this is the first normed database of video clips to include different types of meaningful gestures (emblems and pantomimes) along with meaningless (control) gestures. The features of this database make it suitable for research on neural, cognitive, social, and anthropological aspects of gesture processing.

As of today, the stimuli from the current database have been used to investigate the performance in action recognition and prediction of individuals with upper limb dysplasia (Vannuscorps & Caramazza, 2016) and to investigate the neural representation of gestures in the middle temporal gyrus (Agostini, Papeo, & Lingnau, 2016).

## Method

### Video recording and editing

As a first step, we recorded 278 gestures using a Canon 5D MK2 camera with a frame rate of 24 frames per second and a spatial resolution of 1,920 × 1,088 pixels. The camera was positioned on a tripod approximately 2 m away from the actress. All videos were recorded under the same artificial light condition using a white background, and the actress, a female Italian native speaker, was wearing neutral clothes. The actress was instructed to stand upright with both arms relaxed before performing the gesture, and to return to the same neutral start position after completing the gesture. While performing the gesture, the actress made no noticeable facial expressions (see also Andric et al., 2013; Xu et al., 2009).

**Fig. 1:**
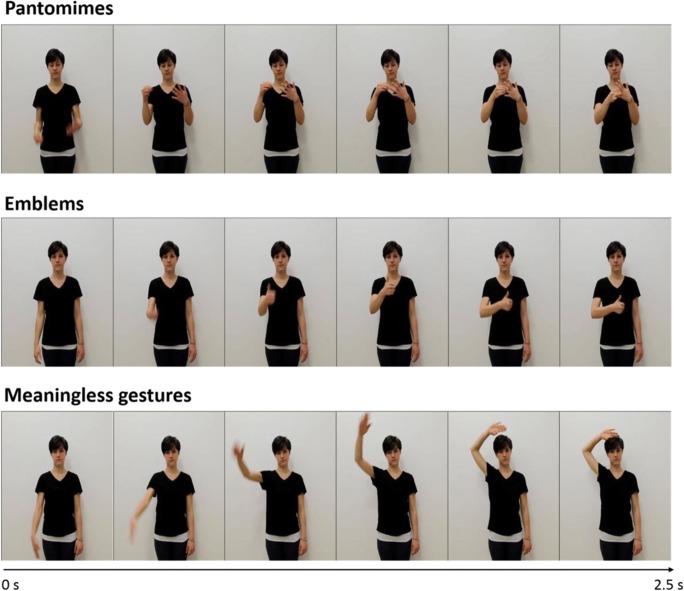
Example stimuli. Still frames from video clips showing pantomimes (i.e., object-directed actions; e.g., *putting on a ring*), emblems (i.e., conventional communicative gestures; e.g., *thumbs up*), and meaningless gestures (i.e., gestures that do not carry any meaning; e.g., *ND_35*). Each video clip lasted 2.5 s

The gestures recorded belonged to three categories: pantomimes (e.g., *putting on a ring*), emblems (e.g., *thumbs up*), and meaningless gestures (see Fig. 1). The selected *pantomimes* were iconic representations of object-directed actions (transitive actions); the *emblems* were conventional gestures, not directed to objects (intransitive actions), but frequently used by Italians to express opinions or elicit behavioral responses. The *meaningless gestures* were hand and arm movements that had no obvious meaning and were created to match the meaningful gestures (pantomimes and emblems) in complexity, form, and pattern of motion. Of the final set of meaningless gestures, 39 were derived from a meaningful gesture (*D* = derived) and 41 were invented from scratch (*ND* = nonderived). In the first case, a spatial feature was changed in order to make a meaningful gesture meaningless. For example, a meaningless gesture was derived from the pantomime for *using binoculars* by having the actress produce the gesture for holding the binoculars and moving them toward her shoulder (as opposed to moving them toward the eyes). As we showed, this spatial modification destroyed the recognizablility of the pantomime.

We compiled an extensive list of emblems and pantomimes commonly used among Italians and recorded them along with the meaningless gestures. The initial set of videos contained 90 pantomimes, 108 emblems, and 80 meaningless gestures. Before recording each video, the actress was instructed on the gesture to perform and was presented with an example (i.e., the same gesture performed by the experimenter). The actress had an unlimited number of trials to produce the gesture as expected.

In a postproduction phase, the videos were cut to a duration of 2.5 s, a time sufficiently long to show the full movement for every gesture. The videos were then resampled to a frame rate of 50 frames per second. The background to the left and right of the actress was cropped, and the sound was removed from the video clips.

### Raters

The 278 video clips were divided into four lists of stimuli (List 1, List 2, List 3, and List 4). Pantomimes, emblems, and meaningless gestures were equally distributed across the four lists. Totals of *N* = 200 Italian raters (137 females, 63 males; mean age = 27.84 years, *SD* = 9.10, range = 18–70) and *N* = 200 American raters (93 females, 107 males; mean age = 39.66 years, *SD* = 11.18, range = 18–70) were randomly assigned to one of the four lists and were divided as follows: (List 1) Italians: *n* = 50, 35 females; Americans: *n* = 50, 19 females; (List 2) Italians: *n* = 50, 36 females; Americans: *n* = 50, 25 females; (List 3) Italians: *n* = 50, 34 females; Americans: *n* = 50, 21 females; (List 4) Italians: *n* = 50, 33 females; Americans: *n* = 50, 27 females. Ten Italian raters and 14 American raters were excluded from the study due to incomplete rating responses. The raters gave informed consent before participating in the study. The study was approved by the Ethics Committee for research involving human subjects of the University of Trento, and it conforms to the Declaration of Helsinki. The first *N* = 60 Italian raters completed the rating without payment, whereas the remaining *N* = 140 Italian raters received €4 for their participation. The American raters received $3 for their participation.

### Procedure

For each item in the four lists, raters were asked to (1) rate the meaningfulness of the gesture, (2) name the gesture, and (3) verbally describe the gesture. Data were collected using an online rating site called Qualtrics (http://www.qualtrics.com/). For Americans, the rating was administered through Amazon Mechanical Turk (https://www.mturk.com/). To ensure the inclusion of reliable raters, only raters with at least a 95% approval rate in previous surveys were included. Since Mechanical Turk is not widely used in Italy, the rating for Italians was administered via email. The Italian participants were recruited using the Center for Mind/Brain Sciences (CIMeC) Facebook page.

In each trial, a video clip was presented at the upper part of the screen on a black background, followed by the three tasks:

1. ***Meaningfulness rating*** Raters were asked to indicate the meaningfulness of each gesture on a 7-point Likert scale (1 = *not meaningful at all*, 7 = *very meaningful*). When the gesture was rated as meaningless (i.e., a rating of “1”), the first *N* = 60 Italian raters and the first *N* = 80 American raters were instructed to skip to the next video clip. To minimize the possibility that participants would assign a rating of 1 to every item, just to complete the survey as quickly as possible, the remaining raters were presented with two multiple-choice questions regarding visuospatial aspects of the video clips. Specifically, raters had to judge (1) the direction of the hand movement (vertical, horizontal, or both directions) and (2) the hands used to perform the gesture (left, right, or both). No differences in ratings were observed between the first and the second *group of raters.
2. ***Naming task*** Raters were asked to name each gesture by typing its name. They were instructed to use only one or two words, if possible. This task was designed to check the comprehensibility of the gestures (i.e., the correspondence between the intended meaning and the meaning assigned by the participants) and the consistency across participants of the interpretation and labeling of the gesture.
3. ***Description*** Raters were asked to provide a description of the meaning of the gesture. The aim of this task was to examine the consistency across participants of the interpretation of the gesture.

At the beginning of each survey, a video clip was presented as an example, for each of the three stimulus types (pantomimes, emblems, and meaningless gestures). The videos used as examples were not included in the final dataset. Raters did not have any time restriction and were allowed to watch a video as many times as needed.

### Data analysis

#### Meaningfulness rating

For the analysis of each video clip, we carried out the following steps: (1) We performed outlier removal by calculating the *z* value for each response and excluding ratings above or below two standard deviations from the mean rate assigned to that video clip; (2) the meaningfulness of each item was computed as the mean for all raters, except for those discarded in Step 1; (3) we also calculated the median of the meaningfulness ratings; (4) gestures with a median >5 were considered to be meaningful gestures, and gestures with a median <3 were meaningless.

#### Naming and description

For each item, we determined: (1) the most frequent meaning and (2) two measures of meaning agreement—namely, (a) the percentage of raters who produced the most frequently provided meaning and any alternative meanings, and (b) Shannon’s diversity index (*H*). The first measure was calculated as the percentage of raters who provided a given meaning. The *H* index was calculated on the basis of the following formula:

$$ {H}^{\prime }=-\sum \limits_{i=1}^R{p}_i\ln {p}_i, $$

where *R* is the number of unique meanings provided and *p*_*i*_ is the proportion of raters who produced each unique meaning. This index provides the dispersion of the different meanings: the *H* index is zero when there is maximum agreement—that is, if the same meaning was provided by all respondents (i.e., by the participants who rated the meaningfulness of the item as higher than 1 and then provided a description of the gesture). The *H* index increases with the increase in the number of alternative responses.

#### Correspondence between intended and rated meanings

Because the meaningfulness rating provides only an indirect measure of the familiarity of gestures, we combined this measure with the responses from the naming task in order to obtain an indication of whether the meaning that a participant assigned to a gesture was the same as the intended meaning. In communication, the task of the addressee is to understand the message as well as to recognize the communicator’s intended meaning. The meaningful gestures (median > 5) were subdivided into three categories: (1) the *correct meaning*, if the most frequent response in the naming task was given in at least 50% of the responses (% > 50) and corresponded to the intended meaning of the gesture, as originally established by the experimenters; (2) a *disagreement*, if the most frequent response in the naming task was the same as the intended meaning, but it accounted for less than 50% of the participants (% < 50); and (3) a *different meaning*, if the most frequent response was different from the intended meaning.

#### Pantomime naming: actions versus objects

The pantomimes in the current dataset were gestures that involved the use of an object. We observed that many raters, when asked to name a gesture, reported the name of the object instead of the name of the action. We analyzed the percentage of raters who provided a verb (denoting the action), a noun (denoting the object), or another grammatical form (adjectives, adverbs, sounds, or short sentences) in the naming task. Since the name of the object and the action are often the same in English, this additional analysis was carried out only on the data from the Italian raters.

#### Emblem naming: events versus states

The emblems in the current dataset can denote actions (e.g., *to clap*) or physical/psychological states (e.g., *thumbs up* to mean agreement). On the basis of the naming and verbal description, we distinguished between emblems that denoted an action (emblems–event, *EE*) and emblems that denoted a state (emblems–state, *ES*). We added this distinction because we considered this information relevant for studies targeting the semantic distinction between events and states (see Bedny, Caramazza, Grossman, Pascual-Leone, & Saxe, 2008; Papeo & Lingnau, 2015; Peelen, Romagno, & Caramazza, 2012). It is important to note that the categorization of gestures as emblems–event or emblems–state was not established a priori but was based solely on the responses given by the raters. Since the gestures were based on the Italian culture, most emblems had a low agreement on meaning or were not recognized at all by the Americans. For this reason, this additional analysis was also carried out only on the data from the Italian raters.

## Results

### Pantomimes

Ninety video clips representing pantomimes were originally recorded and presented, but one was excluded due to a technical failure. Therefore, the analysis of pantomimes included a total of 89 videos.

#### Italians

**Fig. 2:**
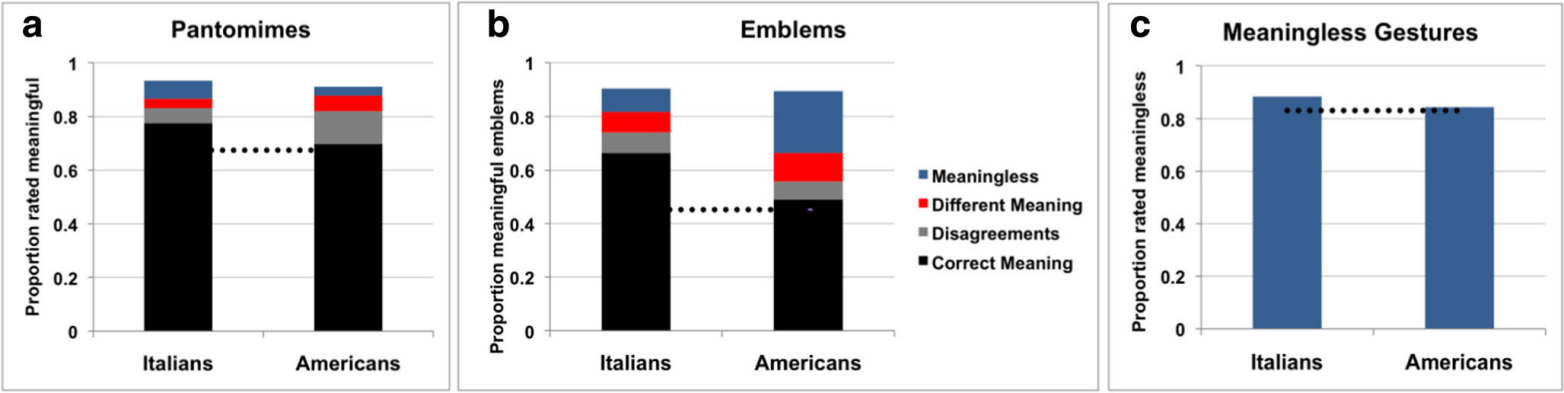
(**a**) Proportions of pantomimes rated as meaningful. Depending on the agreement between the intended meaning of the gesture and the expressed meaning reported in the naming task, the meaningful pantomimes were divided into those given the correct meaning (black), disagreements (gray), different meanings (red), or meaningless gestures (blue) by the Italian and American groups of raters. The horizontal dotted line indicates the proportion of pantomimes that the two groups consistently rated as meaningful and gave their correct meaning. (**b**) Proportions of emblems rated as meaningful. Depending on the agreement between the intended meaning and the expressed meaning that participants reported in the naming task, these were split into those given the correct meaning (black), disagreements (gray), different meanings (red), or meaningless gestures (blue) by the Italian and American groups of raters. The horizontal dotted line indicates the proportion of emblems that the two groups consistently rated to be meaningful and gave their correct meaning. (**c**) Proportions of meaningless gestures rated as meaningless by the Italian and American groups of raters. The horizontal dotted line indicates the proportion of gestures that were consistently rated as meaningless in the two groups

A total of 77 pantomimes were rated as meaningful (median > 5) by the Italian raters. The correct meaning (when the most frequent response in the naming task accounted for at least the 50% of the responses [% > 50] and coincided with the intended meaning of the gesture) was assigned to 69 pantomimes; five of the pantomimes were scored as disagreements (when the most frequent meaning was the same as the intended one, but it accounted for less than 50% of the responses); and three were rated as a different meaning from the intended one (Fig. 2A). Six pantomimes were rated as being meaningless (median < 3).

#### Americans

This group rated 78 pantomimes as meaningful. Of these, 62 pantomimes conveyed the correct meaning, 11 were scored as disagreements, and five were assigned a different meaning in the naming task. Three pantomimes were rated as meaningless (median < 3).

#### Italians and Americans together

Of the total set of 89 pantomimes, 60 were assigned the correct meaning by both groups. Nine gestures were judged as meaningful and were assigned the correct meaning by Italians only (“applying make up,” “blowing a whistle,” “blowing nose,” “opening a lighter,” “playing a cello,” “playing a flute,” “seasoning 2,” “using a hair dryer,” and “using a toothpick”). Two other gestures were judged as being meaningful and were assigned the correct meaning in the naming task by Americans only (“pushing a button” and “shooting with a gun”). Two pantomimes were judged as being meaningless by both groups of raters (“smoking a pipe” and “stamping”).

### Emblems

Of the 108 video clips representing emblems, 104 were included in the final analysis. The rest of the videos were excluded because of technical problems and/or failure to load during the rating.

#### Italians

Of the emblems, 85 were rated as meaningful (median > 5) by the Italian group, with a total of 69 videos assigned the correct meaning. Eight of the videos rated as meaningful were scored as disagreements on their meaning. Eight videos were rated with a different meaning. Nine emblems were considered meaningless by the Italian raters (median < 3).

#### Americans

This group rated a total of 69 videos as meaningful. Fifty-one of these videos were assigned the correct meaning, seven were assigned as disagreements, and 11 were given different meanings (see Fig. 2B). Twenty-four videos presenting emblems were considered meaningless by the Americans, with 20 gestures having a median of 1.

#### Italians and Americans together

Forty-six emblems were considered meaningful and had the correct meaning assigned by both groups. The Italian group only rated another 23 emblems as being meaningful and assigned them the correct meaning: six of these emblems were emblem–events, and 17 were emblem–states. Five different emblems were meaningful with the correct meaning only for the American group. Five emblems were rated as meaningless by both groups.

### Meaningless gestures

Of the 80 video clips of meaningless gestures, 77 were included in the final analysis. Three gestures were excluded prior to testing because of unintended similarity to potentially offensive gestures.

#### Italians

A total of 68 gestures were rated as meaningless (median < 3) by the Italian group. For 66 of those gestures the median was as low as 1, indicating that the majority of these gestures indeed did not resemble any familiar gestures for the Italian group.

#### Americans

This group rated 66 of the videos as meaningless (median < 3), out of which 64 gestures had a median of 1 on the meaningfulness scale.

#### Italians and Americans together

Both groups consistently rated 64 of the videos in this category as meaningless (see Fig. 2C).

### Datasets

In the dataset, we report the collected norms for 89 pantomimes, 104 emblems, and 77 meaningless gestures, for Italians (see Table S1) and Americans (see Table S2). For each item, we provide the following information: (a) the position in the table with respect to the other items, depending on their rating (*Position*); (b) the English name/description of the item, as intended during the recording (*Item*), which is also the name under which the video appears in the database; (c) the mean and median of the meaningfulness ratings, rated on a Likert scale from 1 to 7; (d) the most frequently produced meaning; (e) the agreement on meaning, split by the percentage of raters who introduced the most frequent meaning (*p*), the percentage of raters who judged the gesture as being meaningless (i.e., ratings of 1 on the meaningfulness scale) and did not provide a description (*NA*), and meaning agreement (*H* index); (f) the percentages of raters who named the gesture with a verb, with a noun, or with another grammatical form (pantomimes rated by Italians only—see the Pantomime Naming: Actions vs. Objects section); (g) for emblems only, the mapping of the gestures onto an event (emblem–events, *EE*) or a state (emblem–states, *ES*), as intended during the recording (emblems rated by Italians only—see the Emblem Naming: Events vs. States section); and (h) alternative meanings and their percentages of agreement. In a few cases, the same rater provided two or more names for the same video (e.g., *screwing or unscrewing*). We considered both names different answers; therefore, the sum of the percentages of these particular items was higher than 100. In some cases, the majority of raters provided a different meaning from the intended one. We mark these gestures with two asterisks next to the gesture’s name in each table.

Pantomimes and emblems are ordered so that the meaningful gestures are at the top of the table, starting from the ones with the highest agreement on the correct meaning (highest %). Gestures with the same mean ratings are listed in alphabetical order. For the Italian group, the meaningfulness rating yielded a set of 77 meaningful pantomimes (see P1–P77 in Table S1): 69 correct meaning pantomimes (P1–P69 in Table S1), five disagreement pantomimes (P70–P74 in Table S1), and three different meaning pantomimes (P75–P77 in Table 1); there were also six meaningless pantomimes (P83–P86 and P88–P89).

For the American group, the ratings identified 78 meaningful pantomimes (see P1–P78 in Table S2): 62 correct meaning pantomimes (P1–P62 in Table S2), 11 pantomimes with disagreement (P63–P73 in Table S2), and five different meaning pantomimes (P74–P78 in Table S2). Three pantomimes were rated as meaningless (P86, P88 and P89).

For the emblems, Italians identified 85 meaningful emblems (E1–E85 in Table S1): 69 correct meaning emblems (E1–E69 in Table S1), eight emblems with disagreement (E70–E77 in Table S1), and eight emblems with a different meaning (E78–E85 in Table S1). They rated nine emblems as meaningless (E93–E98 and E102–E104). The American group considered 69 emblems to be meaningful (E1–E69 in Table S2): 51 correct meaning gestures (E1–E51 in Table S2), seven with disagreements (E52–E58 in Table 2), and 11 with a different meaning (E59–E69 in Table 2). The Americans rated 25 emblems as meaningless (E76–E78 and E83–E104).

The tables for meaningless gestures are arranged with the gestures rated as meaningless at the top (median < 3), in ascending order, based on the mean of the meaningfulness rating. Gestures with the same mean ratings are listed in alphabetical order. Italians rated 68 meaningless gestures as meaningless (M1–M68 in Table S1), whereas Americans found 66 of them to be meaningless (M1–M66 in Table S2).

## Discussion

The aim of this study was to develop and make publicly available a library of video clips of symbolic gestures (pantomimes and emblems), and matched meaningless gestures. This database has been conceived to be used in a wide range of research areas addressing the processing of meaningful manual gestures, including neuroimaging studies on the neural basis of symbolic gesture recognition, research on integration of manual and symbolic aspects of communicative gestures, on the contribution of culture in shaping and understanding pantomimes and emblems, as well as on the selective impairment of some aspects of gestural production or comprehension in brain-damaged patients. This library has several advantages. The first one is its size: it provides norms for pantomimes, emblems, and meaningless gestures for a total of 270 gestures. Second, it provides high-quality videos of symbolic gestures controlled for a number of factors: the same actress performs all the gestures, the facial expression is neutral and comparable across all the videos, the background is neutral and identical across videos, all videos have the same length and were recorded and edited with the same equipment and software packages. Third, the videos were rated by a large sample of raters in a set of tasks (meaningfulness score, naming and description) designed to obtain a reliable index of the meaningfulness of the gestures and of the correspondence between the intended meaning as initially established by the experiments and the expressed meaning (i.e., *the meaning as understood by the raters*). Fourth, the sample included two groups of raters, belonging to different cultural groups (Italians and Americans), which offered insight on potential cross-cultural variations in understanding the current gestures. This library, available for non-commercial purposes via the database Figshare (10.17637/rh.c.4219988), aims to encourage and facilitate research on symbolic gestures, but also to promote replicability of results among studies.

The present study yielded a number of observations, highlighting important distinctions within the broad category of symbolic gestures. First, we found higher consistency between Italians and Americans in the rating of pantomimes, as compared to the rating of emblems, although both sets of gestures were based on the Italian culture. In particular, we found that Italians and Americans provided consistent judgments for most pantomimes (the 67% of pantomimes were considered meaningful and interpreted as expected by both groups). In contrast, only the 45% of the emblems were considered meaningful and were assigned the correct (intended) meaning, consistently by both groups. Pantomimes are object-directed actions that are not constructed on the basis of cultural conventions, but rather by reproducing the real-world referent (i.e., the object-directed action; van Nispen et al., 2017). In the case of emblems, the relationship between meaning and form is culture-specific and can be hardly understood without prior contact with that culture. In our dataset, the higher agreement between Italian and American raters on the interpretation of pantomimes, relative to the interpretation of emblems confirms this distinction (Calbris, 1990; Ekman, 1976; Ekman & Friesen, 1969; Kita, 2009; Payrató, 1993; Poggi & Zomparelli, 1987).

The different susceptibilities of pantomimes and emblems to culture was first investigated from an anthropological perspective (Efron, 1941, 1972; Ekman & Friesen, 1969, 1972; Kendon, 1992; Poggi & Zomparelli, 1987; Payrató, 1993). It has been suggested that the learning of emblems typical of a culture and the learning of words are achieved in a similar fashion, by learning an association between a form and its shared meaning (Ekman, 2004; Ekman & Friesen, 1969; Gullberg, 2006). The observation that emblems are similar in nature to words has been corroborated by neuroscientific investigation with event-related potentials, which has shown that the difference in the neural activity between emblems and meaningless gestures is analogous to the difference observed between words and pseudowords (Gunter & Bach, 2004; Wu & Coulson, 2005). Just like words, emblems have been suggested to be susceptible to cultural influence, and more so than pantomimes. The present results support this circumstance, showing that Americans performed differently from Italians, and they did even more so with emblems than with pantomimes. In many cases, Americans assigned a different meaning or no meaning to the Italian emblems. Thus, our observations highlight the culture-specific arbitrary pairing between an emblematic gesture and its meaning, in two ways: (1) Some gestures can result completely opaque to members of other cultures (high rate of meaningless emblems for Americans; e.g., the Italian emblem for “full of people” had no significance for Americans; Calbris, 1990), and (2) the same emblematic gesture can take a different meaning in different cultures (the Italian emblem for “quietly” was interpreted as “stop” by Americans; Morris, 1979). Yet, Americans could correctly comprehend a subset of the Italian emblems. The emblems correctly identified by both Americans and Italians could represent a set of symbolic gestures that two Western cultures share, possibly due to their cultural similarities and contact in the recent and more distant past (McClave, Kim, Tamer, & Mileff, 2007; Morris, 1979).

Interestingly, the emblems that were interpreted consistently across cultures appear to be those that denoted bodily actions such as *sleeping* or *yawning* (see also Ekman & Friesen 1972). Those bodily actions are subject by the anatomy of the body and therefore are performed in a similar manner by all human beings, and when used as emblems (e.g., to communicate fatigue), they are characterized by high degree of iconicity. In contrast, higher disagreement between the two groups applied to emblems with a more arbitrary relation between form and meaning. For example, the emblem for “tasty,” in which the actress points the index finger to her cheek and slightly rotates the hand, was easily recognized by all Italians with 100% of agreement, but it was judged as meaningless by the American raters.

Overall, our dataset emphasizes the variability of individuals’ interpretations of pantomimes and—to a greater extent—of emblems. This variability is such that the same gesture (e.g., *yawning*) can be named with a verb (“to yawn”) or a noun (“the yawn”) or can be mapped on different meanings, on the basis of which it could fall in one gesture category or another (e.g., the pantomime for “yawning” becomes an emblem if it is interpreted as “I am bored”). Acknowledging this variability, the present work identifies statistical regularities at the population level (one gesture is more frequently interpreted as X than Y), rather than strict categorical distinctions (one gesture is X and not Y).

Finally, 83% of the meaningless gestures were consistently rated as meaningless by Italians and Americans alike, showing that this set of videos can serve as a meaningless control condition in behavioral and neuroimaging studies.

In conclusion, we provide 270 highly controlled quality videos of pantomimes, emblems, and meaningless gestures, together with the results of a large-scale rating study to establish their meanings and consistency across individuals and cultural groups. We hope that this database can stimulate research on the processing of gestures and can promote the replication of observations from independent studies.

## Electronic supplementary material

ESM 1 (PDF 71.7 kb)

ESM 2 (PDF 69.0 kb)
