# Supplementary material for: A norming study of high-quality video clips of pantomimes, emblems, and meaningless gestures
Source: Behav Res Methods. 2018 Dec 12;51(6):2817–26. doi: 10.3758/s13428-018-1159-8 (PMC6877486; doi:10.3758/s13428-018-1159-8)
Supplement: Supplementary file 1 — (PDF 71.7 kb) [file 13428_2018_1159_MOESM1_ESM.pdf]

Table 1 Statistical analysis carried out on pantomimes, emblems, and meaningless gestures (Italian group of raters)

Position	Item's Name	Meaningfulness		Most Frequent Meaning	Meaning Agreement			Verb	Noun	Other Form	Alternative Meanings and Percentages of Agreement
		Mean	*Median*		*%*	*NA*	*H*				
PANTOMIMES (ITALIAN RATERS)											
P1	drinking	7.00	7	drinking	100	0	0.00	9	91		
P2	playing basketball	7.00	7	playing basketball	100	0	0.00	70	30		
P3	pouring from a bottle	7.00	7	pouring	100	0	0.00	12	88		
P4	smoking a cigarette	7.00	7	smoking	100	0	0.00	23	77		
P5	turning pages	7.00	7	turning pages	100	0	0.00	15	83		
P6	driving	6.95	7	driving	100	0	0.00	30	70		
P7	playing flute	6.95	7	playing flute	100	0	0.00	43	57		
P8	playing guitar	6.93	7	playing guitar	100	0	0.00	64	36		
P9	reading	6.93	7	turning pages/reading	100	0	0.00	14	86		
P10	taking pictures	6.93	7	taking a picture	100	0	0.00	38	60		
P11	combing 1	6.89	7	combing hair	100	0	0.00	9	91		
P12	putting on a ring	6.87	7	putting on a ring	100	0	0.00	59	41		
P13	putting on earrings	6.87	7	putting on earrings	100	0	0.00	42	36		
P14	putting on glasses	6.84	7	putting on glasses	100	0	0.00	55	45		
P15	rocking a baby	6.84	7	rocking a baby	100	0	0.00	26	74		
P16	filing nails	6.83	7	filing nails	100	0	0.00	31	69		
P17	driving a motorbike	6.77	7	driving/starting a motorcycle	100	0	0.00	50	50		
P18	calling on a telephone	6.76	7	calling on a telephone	100	0	0.00	46	54		
P19	playing golf	6.75	7	playing golf	100	0	0.00	77	23		
P20	spraying perfume	6.75	7	spraying perfume	100	0	0.00	43	57		
P21	writing	6.74	7	writing	100	0	0.00	14	86		
P22	using a mobile phone	6.64	7	using a mobile phone	100	0	0.00	40	60		
P23	knotting	6.59	7	tying something	100	0	0.00	46	54		
P24	zipping up a jacket	6.53	7	zipping up a jacket	100	0	0.00	24	53		
P25	whipping	6.44	7	whipping/stirring	100	0	0.00	12	88		
P26	eating with a spoon	6.40	7	eating with a spoon	100	0	0.00	16	84		
P27	sweeping	6.38	7	sweeping	100	0	0.00	17	83		
P28	combing 2	6.23	7	combing hair	100	0	0.00	19	81		
P29	throwing	6.22	7	throwing something	100	0	0.00	30	70		
P30	eating with a fork	6.07	7	eating	100	0	0.00	14	86		
P31	using binoculars	6.65	7	looking through binoculars	98	0	0.10	42	58		

Table 1 (continued)

P32	blowing nose	6.55	7	blowing nose	98	0	0.10	21	79					
P33	washing hands	6.31	7	opening the tap to wash the hands	98	0	0.11	22	78					
P34	painting a wall	6.02	7	painting	98	0	0.11	23	77					
P35	using a remote control	6.17	7	using a remote control	98	0	0.11	54	46					
P36	spraying deodorant	6.53	7	spraying deodorant	96	0	0.21	80	20					
P37	playing violin	6.89	7	playing violin	95	0	0.18	43	57					
P38	playing drums	6.47	7	playing drums	95	0	0.22	58	42					
P39	using a corkscrew	6.33	7	removing the cork	95	0	0.22	33	67					
P40	spraying	6.08	7	spraying/writers	95	0	0.25	54	46					
P41	smoking a cigar	6.54	7	smoking	93	0	0.32	22	78					
P42	putting on a necklace	6.76	7	putting on a necklace	91	0	0.30	54	46		putting on a bib (9)			
P43	changing a light bulb	6.22	7	changing a light bulb	91	0	0.30	41	59		screwing/twisting something (9)			
P44	turning a key	6.47	7	turning a key	88	0	0.36	40	60		turning/twisting something (12)			
P45	peeling a banana	6.18	7	peeling a banana	84	0	0.50	32	66	2	opening something (14)			
P46	applying make up	5.09	6	applying make up	81	0	0.50	35	65		combing hair (22)			
P47	stirring	5.11	6	stirring/mixing	80	16	0.24	5	95					
P48	uncorking a bottle	6.36	7	opening a bottle	79	0	0.67	23	77		opening a jar/lid (23)			
P49	archery	6.77	7	archery	79	0	0.69	60	37		using a slingshot (14)			
P50	using a toothpick	5.20	6	using a toothpick	76	22	0.13	67	33					
P51	seasoning 1	4.82	5	seasoning	76	18	0.27	35	65		pouring (6)			
P52	opening a jar 1	6.64	7	opening a jar	75	0	0.58	100			closing a jar (30)			
P53	dealing cards	6.44	7	dealing cards	74	0	0.66	37	63		handing out papers (26)			
P54	sewing	4.48	5.5	sewing	74	24	0.13	17	83					
P55	seasoning 2	4.16	5	seasoning	73	24	0.12	22	78					
P56	ironing	4.09	5	ironing	72	0	0.89	14	86		pouring something (17)			
P57	painting nails	4.65	5	painting nails	71	29	0.00	71	29					
P58	wiping the mouth	4.61	5	wiping the mouth	71	24	0.25	16	84					
P59	opening a jar 2	6.44	7	unscrewing	70	0	0.70	19	81		screwing (33)			
P60	cutting with a knife	4.70	6	cutting/chopping	70	30	0.00	25	75					
P61	fastening a security belt	5.41	7	seatbelt	65	17	0.51	55	45		put something in a bag/pocket (17)			
P62	putting on a belt	4.76	7	putting on a belt	65	30	0.28	69	31					
P63	rowing	4.61	5	rowing	63	20	0.78	3	97		biking (6)			
P64	playing cello	6.67	7	playing cello	63	0	1.16	46	54		playing violin (15)	playing bass (11)	playing a music	scratching hair (7)

Table 1 (continued)

													instrument (7)
P65	applying lipstick	4.74	6	applying lipstick	63	28	0.44	73	27		indicates to keep the mouth shut (7)		
P66	blowing a whistle	6.34	7	blowing a whistle	63	0	1.11	51	49		coughing (26)	using a breathalyzer (7)	
P67	typing	6.54	7	typing on a keyboard	63	0	0.66	40	60		playing piano (44)		
P68	using a hair dryer	4.47	5	drying hair	58	0	1.03	50	50		spraying hairspray (25)	washing hair (14)	
P69	opening a lighter	4.57	5	opening a lighter	57	24	0.68	70	27	3	using a remote control (14)		
P70	grinding pepper	5.93	7	grinding pepper	45	2	1.20	24	76		opening a jar (31)	closing a jar (21)	
P71	playing cards	4.52	5	dealing cards	41	26	1.23	44	56		taking something out and giving it to someone (13)	paying (11)	dividing something (7)
P72	pushing a button	6.33	7	pushing a button	38	0	1.09	38	58	5	ringing the bell (35)	pointing at someone or something (28)	
P73	washing hair	5.55	6	washing hair	26	0	1.43	49	51		fixing hair (44)	indicates being nervous (19)	massaging to hair (9)
P74	hammering	3.88	5	hammering	24	39	1.56	30	67	3	knocking (16)	tapping/hitting (8)	blessing (6)
P75	erasing**	5.95	6	writing/ scribbling	63	0	1.05	10	90		erasing (20)	painting (12)	
P76	screwing**	6.02	7	turning a key	60	0	1.01	31	67		screwing (31)		
P77	cutting with scissors**	4.41	5	stapling	50	37	0.59	24	76		cutting with scissors (11)		
P78	wiping	3.78	4	wiping/ cleaning	49	47	0.49	21	5				
P79	putting on a watch	3.76	4	putting on a watch	43	31	0.83	25	8		putting on a bracelet (39)		
P80	playing tennis	3.63	4	playing tennis	37	43	0.77	10	16		throwing something (18)		
P81	lighting a match	3.53	3	lighting a match	29	47	1.22	19	6	1	ripping a paper (10)	checkmark (10)	
P82	folding fun	3.59	3	using a fan	27	47	1.12	16	15		using a spray (16)	using a hairdryer (8)	
P83	stamping	3.43	2	stamping	47	42	0.69	13	13				
P84	shooting with a gun	3.06	1	shooting with a gun	43	57	0	5	15				
P85	sawing	2.70	1	cutting/ slicing	33	59	0.73	11	8				
P86	eating an apple	3.13	1	peeling	31	59	0.80	20	0		unwrapping (6)		
P87	measuring**	4.00	4	opening a zip	47	29	0.80	23	12		measuring with a meter (20)		
P88	smoking a pipe**	1.88	1	blowing	9	76	1.47	11	0		smelling (7)		

Table 1 (continued)

P89	screwing**	1.89	1	seasoning	9	74	1.63	8	3	1	unscrewing (6)
Position	Item's Name	Meaningfulness		Most Frequent Meaning	Meaning Agreement			Emblem Event	Emblem State	Alternative Meanings and Percentages of Agreement	
		Mean	*Median*		*%*	*NA*	*H*				
EMBLEMS (ITALIAN RATERS)											
E1	calling	7.00	7	calling	100	0	0.00	x			
E2	cutting throat	7.00	7	threatening to death	100	0	0.00		x		
E3	no	7.00	7	no	100	0	0.00		x		
E4	silence	7.00	7	"silence!"	100	0	0.00		x		
E5	sleeping	7.00	7	being sleepy/needing sleep	100	0	0.00	x			
E6	tasty	7.00	7	indicates that a food is good	100	0	0.00		x		
E7	waving hello 1	7.00	7	saying hi	100	0	0.00	x			
E8	waving hello 2	6.98	7	saying hi	100	0	0.00	x			
E9	counting	6.96	7	count to five	100	0	0.00	x			
E10	I kill myself	6.94	7	shoot me!	100	0	0.00	x			
E11	kiss	6.93	7	blowing a kiss	100	0	0.00	x			
E12	writing	6.93	7	writing	100	0	0.00	x			
E13	come here	6.91	7	asking someone to come closer	100	0	0.00	x			
E14	pointing	6.91	7	to point at a person	100	0	0.00	x			
E15	money	6.84	7	indicates money	100	0	0.00		x		
E16	ok	6.84	7	ok, perfect	100	0	0.00		x		
E17	clapping	6.81	7	to clap	100	0	0.00	x			
E18	fear	6.80	7	indicates fear	100	0	0.00		x		
E19	four	6.80	7	*four* as a quantity	100	0	0.00		x		
E20	salute	6.80	7	salute	100	0	0.00	x			
E21	sending away	6.80	7	kicking someone away	100	0	0.00	x			
E22	three	6.80	7	*three* as a quantity	100	0	0.00		x		
E23	strangling	6.79	7	strangling metaphorically	100	0	0.00	x			
E24	gross smell	6.78	7	indicates a gross smell	100	0	0.00		x		
E25	eating	6.74	7	being hungry and requiring some food	100	0	0.00		x		
E26	I shoot at you	6.73	7	indicates the intention of killing	100	0	0.00	x			
E27	approving	6.67	7	approving	100	0	0.00		x		
E28	walking	6.67	7	to walk	100	0	0.00	x			
E29	shouting	6.62	7	shouting/ calling aloud	100	0	0.00	x			
E30	I beat you	6.47	7	intimidating someone	100	0	0.00	x			
E31	bowing	6.14	7	bowing	100	0	0.00	x			
E32	swearing	6.14	7	swearing not to talk	100	0	0.00		x		
E33	disapproving	6.02	7	disapproving	100	0	0.00		x		
E34	coupling	5.93	7	indicates union between persons or objects	100	0	0.00		x		
E35	finger crossed	6.87	7		98	0	0.10		x		

Table 1 (continued)

				finger crossed/ express good luck							
E36	speaking	6.11	7	indicates someone is speaking too much	98	0	0.10	x			
E37	looking far	6.39	7	looking far away	98	0	0.11	x			
E38	yawning	6.81	7	yawning because of tiredness or boredom	98	0	0.11	x			
E39	crazy 2	6.25	7	"are you crazy?!"	98	0	0.11		x		
E40	I don't care	6.78	7	I don't care/ indifference	96	0	0.18		x		
E41	run away	6.69	7	driving away/shoo someone	96	0	0.18	x			
E42	crazy 1	6.47	7	indicates that a person is crazy	96	0	0.18		x		
E43	slowing down	6.79	7	slowing down/keeping calm	95	0	0.22	x			
E44	arresting	6.38	7	indicates the detention of someone	95	0	0.20	x			
E45	caress	5.84	7	caressing	93	0	0.24	x		cleaning the face (7)	
E46	forgetting	6.60	7	realizing something that was forgotten	93	0	0.30		x		
E47	stop	6.40	7	stop	93	0	0.30	x			
E48	stomach ache	6.12	7	having a stomach ache	91	0	0.31			bowing (9)	
E49	triumphing	5.91	6	triumphing	88	0	0.49		x	gesture of communism (10)	
E50	what do you want?	6.63	7	"what do you want? What are you saying?"	88	0	0.43		x	indicates disagreement (10)	
E51	quietly	6.93	7	"wait! Be quiet"	84	0	0.53		x		
E52	long time ago	6.36	7	indicates something has already happened time ago and now it's too late	80	0	0.68		x	"forget about it" (11)	
E53	idea	5.22	6	getting an idea	80	20	0.00		x		
E54	drinking	5.33	7	ask for a drink	79	19	0.12	x			
E55	so-so	5.61	6	so-so/indicates uncertainty	76	0	0.55		x	almost (24)	
E56	cutting	6.67	7	to call for shorten what a person is doing (speaking, . . .)	74	0	0.59	x		symbol of scissors (30)	
E57	praying 1	6.35	7	praying someone	74	0	0.79	x		greetings/ thank you in Japanese (19)	peace (12)
E58	listening	6.74	7	to listen with more attention	72	0	0.59	x		indicates that I did not understand (28)	
E59	I don't know	7.00	7	I don't know	68	0	0.63		x	I can't do anything—I don't care (34)	
E60	finished	6.20	7	indicates that something is finished/there is nothing anymore	68	0	0.60		x		
E61	two	6.52	7	*two* as a quantity	65	0	0.82		x	victory (35)	peace (7)

Table 1 (continued)

E62	unpleasant	4.13	5	finding someone/ something unpleasant	64	29	0.31		x	indicates that the food was not digested well (7)			
E63	being late	6.85	7	indicates that is late	63	0	0.86		x	asking “what’s the time” (48)	indicating the time (9)		
E64	later	4.76	5	communicates to postpone something	63	15	0.73		x	asking to repeat (15)	indicates to go on (7)		
E65	crazy 3	6.82	7	indicates someone is crazy	60	0	0.67		x	thinking/ using the brain (40)			
E66	cunning	5.00	6	indicates astuteness	58	25	0.91		x				
E67	loser	4.55	5	calling someone a loser	58	35	0.38		x				
E68	anger	6.54	7	indicates anger	58	0	1.29		x	yawning (23)			
E69	getting tired	6.76	7	express effort in doing something	52	0	0.69		x	express relief (48)			
E70	closer	4.30	5	asking someone to come closer	48	24	0.86	x		kicking away (24)			
E71	hitchhiking	5.57	6	hitchhiking	48	0	1.03	x		indicates someone/something on the right (35)	indicates a direction “over there” (18)		
E72	sated	6.33	7	being full in the stomach	39	0	1.32		x	bring hungry (46)	indicates having a stomach ache (11)	massaging the belly (9)	
E73	smelling	4.59	5	smelling something	38	17	1.54	x		indicates a bad smell (19)	indicates astuteness (15)	to suspect something (6)	sniffing cocaine (6)
E74	to bear a grudge	4.45	6	indicates that I will remember something bad that happened	37	12	1.09		x	tying (16)	indicates later (12)		
E75	horns	6.12	7	symbol of horns	37	0	1.35		x	sign of rock ’n roll (24)	indicates someone that was cheated (22)	superstitious gesture, against the bad luck (17)	
E76	I don’t know anything	6.16	7	I don’t know	35	0	0.88		x	It wasn’t me (63)	backing off from a situation (9)		
E77	praying 2	5.13	6	begging someone	31	15	1.19	x		praying (31)	indicates togetherness (27)		
E78	congratulations**	5.98	7	having an agreement	64	0	1.10		x	greetings (14)	being together (14)	congratulations (7)	
E79	looking at**	6.40	7	checking/ keeping an eye on	59	0	0.68	x		watching (41)			
E80	rub one’s hands**	6.13	7	washing hands	56	0	1.23	x		showing satisfaction (22)	foretaste of victory (13)	indicates the intention of doing something that seems smart (9)	
E81	standing up**	5.84	6	asking to rise something (volume/ intensity)	50	0	1.25	x		asking to rise an object (34)	inviting someone to stand up (32)		
E82	a moment**	4.92	5	*one* as a quantity	49	0	1.05		x	asking for a moment (33)	asking for attention/ for speaking (23)		
E83	dying**	6.39	7	indicates that something is over	43	0	1.30		x	indicates that someone is dead (41)	cross symbol (9)		

Table 1 (continued)

E84	agreement**	6.52	7	paying attention	34	0	1.65		x	indicates astuteness (30)	keeping an eye on someone (18)	looking (7)	
E85	winning**	6.07	7	*two* as a quantity	32	0	1.36		x	victory (52)	peace (11)	indicates that everything is fine (7)	
E86	getting crazy	3.82	4	getting crazy	43	39	1.11		x	screwing something (6)			
E87	stealing	3.94	4	indicates stealing something	39	39	1.22	x					
E88	putting together	3.90	4	connecting/ joining	27	35	1.45	x		shortening (22)	indicate something small (6)		
E89	thinking	3.96	4	thinking	26	41	1.54	x		having in mind (13)	being crazy (9)		
E90	exploding 2	3.14	3	explosion	44	38	1.00	x		surprise (6)	magic trick (6)		
E91	lowering	3.27	3	lowering	43	45	0.83	x					
E92	union	3.52	3	indicate a strong union/ relation	28	43	1.32		x	promise (15)	being connected (7)		
E93	skinny	3.35	2.5	being thin	37	50	0.92		x				
E94	being alone	2.87	2	asking “so what?,” indicate something vague	33	59	0.73		x				
E95	hungry	3.18	2	to be hungry	20	49	1.79		x	stomach ache (6)	that’s enough (6)	indicate the height of something (6)	
E96	inverting	1.78	1	flipping over an object	20	76	0.60	x					
E97	growing up	2.70	1	growing up	20	59	1.11	x		rising (15)			
E98	I care about you	3.00	1	being close to someone/ showing love	20	57	1.47		x	congratulation (13)			
E99	good guy**	3.76	4.5	supporting/ encouraging	30	37	1.20	x		compliments (22)			
E100	snapping fingers	4.43	4.5	snapping fingers	22	28	1.76	x		doing something quickly (13)	what a pity! (13)	asking for attention (11)	keeping the rhythm (7)
E101	begging**	3.93	4	offering	43	28	0.94	x		begging (15)	showing something (13)		
E102	exploding1**	2.96	2	breaking/ dividing	28	54	1.19	x		stretching (9)			
E103	full of people	1.57	1	full of people	11	82	0.90		x				
E104	collision**	2.17	1	two things getting together	24	52	1.55		x	indicate uncertainty (7)			
		Meaningfulness		Most Frequent Meaning	Meaning Agreement			Alternative Meanings and Percentages of Agreement					
Position	Item’s Name	*Mean*	*Median*		%	*NA*	*H*						
MEANINGLESS (ITALIAN RATERS)													
M1	D_28 (using a hair dryer)	1.00	1		0	100							
M2	ND_07	1.00	1		0	100							
M3	ND_14	1.00	1		0	100							
M4	ND_37	1.00	1		0	100							

Table 1 (continued)

M5	D_33 (screwing)	1.02	1			95		
M6	ND_17	1.02	1		0	100		
M7	D_16 (sweeping)	1.04	1			100		
M8	ND_18	1.04	1		0	100		
M9	ND_29	1.04	1			100		
M10	ND_23	1.05	1			98		
M11	ND_33	1.05	1			95		
M12	D_26 (walking)	1.07	1			95		
M13	ND_15	1.07	1			98		
M14	ND_35	1.07	1			95		
M15	D_27 (praying 2)	1.09	1			95		
M16	ND_19	1.12	1			93		
M17	ND_20	1.12	1	touching someone on the shoulder	9	91	0.00	
M18	ND_21	1.12	1			95		
M19	ND_28	1.12	1			98		
M20	D_10 (praying 1)	1.13	1			89		
M21	D_22 (combing 2)	1.14	1			93		
M22	D_11 (opening a lighter)	1.18	1			91		
M23	D_14 (smoking)	1.18	1			93		
M24	D_30 (writing 2)	1.19	1			91		
M25	ND_22	1.19	1			93		
M26	D_05 (peeling a banana)	1.20	1			89		
M27	D_32 (sewing)	1.20	1	sewing	7	90	0.00	
M28	D_18 (writing 1)	1.22	1	writing	7	89	0.67	
M29	D_02 (playing flute)	1.25	1	playing an instrument	11	86	0.45	
M30	ND_11	1.26	1			91		
M31	ND_32	1.27	1			93		
M32	ND_13	1.29	1	crashing	11	84	0.80	
M33	D_07 (turning a key)	1.32	1	screwing	7	91	0.56	
M34	ND_30	1.38	1			90		
M35	ND_06	1.39	1			89		
M36	D_23 (cleaning)	1.40	1			88		
M37	ND_02	1.40	1	indicates the number 3	18	78	0.64	
M38	D_04 (cutting with a knife)	1.41	1	using a western lasso	9	82	1.21	
M39	D_25 (using binoculars)	1.41	1	putting something on the shoulder	11	86	0.00	
M40	ND_24	1.42	1			84		
M41	ND_25	1.42	1		0	100		
M42	D_15 (applying lipstick)	1.44	1	applying/fixing make up	16	80	0.53	
M43	D_21 (calling on a telephone)	1.50	1	turning	10	81	1.21	spinning (5)

Table 1 (continued)

M44	ND_10	1.52	1	pointing the top of the nose	14	82	0.74		
M45	D_31 (pouring from a bottle)	1.53	1	tipping over/spilling	13	83	0.00		
M46	D_24 (erasing)	1.54	1	cleaning	12	76	0.61	leveling (10)	
M47	ND_36	1.54	1	joke between kids	10	83	1.01		
M48	D_19 (waving hello)	1.63	1	attracting the attention	7	85	1.00	"can you see me?" (7)	
M49	D_20 (brushing teeth)	1.63	1	hammering/thumbing	12	76	1.17	washing teeth (7)	
M50	ND_26	1.63	1	doing some magic	7	81	0.94		
M51	ND_27	1.64	1	don't want to see	12	79	0.00	saying a secret (7)	
M52	D_08 (sleeping)	1.74	1	sleeping	15	74	1.08		
M53	D_17 (winning)	1.77	1	indicates two persons	17	74	0.64	to blind (9)	
M54	D_37 (listening)	1.77	1	listening	16	77	0.61	putting something on the ear to listen (7)	
M55	ND_04	1.78	1	combining something	20	74	0.84		
M56	D_09 (painting nails)	1.82	1	painting nails	7	80	1.31	writing/ paining on the hand (7)	
M57	ND_09	1.94	1	crashing on something	11	77	1.54		
M58	ND_08	2.06	1	two sides of something	23	68	0.95		
M59	ND_05	2.18	1	stop	20	69	0.99	physical exercise (7)	
M60	D_13 (driving)	2.26	1	opening/closing something over the head (trapdoor, valve, submarine door)	38	60	0.21		
M61	ND_01	2.27	1	indicates come here	25	58	1.03	asking to kiss the hand (10)	
M62	ND_03	2.29	1	drawing a spiral shape	31	56	0.87		
M63	ND_31	2.42	1	indicates togetherness	37	58	0.73		
M64	D_12 (grinding pepper)	2.67	1	unscrewing	22	55	1.16	grinding pepper/salt (10)	screwing something (10)
M65	D_29 (calling)	2.72	1	slang: to greet or cool/ hang loose	22	61	0.69	calling (13)	
M66	D_06 (wearing glasses)	2.82	1	wearing a mask	43	55	0.18		
M67	D_01 (eating with a spoon)	2.96	2	pouring something on the head	33	49	0.88	wearing a hat (12)	scooping (6)
M68	D_03 (combing 1)	3.16	2	cleaning the sleeve of the jacket	37	47	0.89	shaving arms (10)	
M69	ND_12	3.31	3		59	35	0.37		

Table 1 (continued)

				covering eyes not to see something						
M70	ND_40	3.63	3.5	forgetting	26	37	127	checking fever (24)	headache (7)	
M71	ND_16	3.59	4	indicate a lie	27	45	109	making fun of someone/ bantering (20)	getting drunk (6)	
M72	D_35 (stirring)	4.02	4	lasso, cowboy	52	26	84	twirling something (15)		
M73	D_36 (washing hair)	4.15	4.5	rubbing hurt shoulder	37	24	109	massaging (28)	washing/ showering (7)	
M74	ND_39	4.49	5	waving/water motion	63	31	35			
M75	ND_38	4.63	6	removing a ring	71	25	21			
M76	D_34 (triumphing)	5.06	6	indicate strength	29	16	160	threatening someone (27)	indicate anger(12)	showing the fist (8)
M77	ND_34	6.00	6	indicate a sudden change	66	2	112	flipping something (12)	cooking both sides (10)	

** indicates gestures for which the most common meaning was different from the expected one

For meaning agreement:

% = percentage;

NA = no meaning (percentage of participants that did not provide a meaning, i.e., rated the gesture as meaningless);

H = Shannon's diversity index (the *H* value increases as the naming agreement decreases; $H = 0$ means that only one meaning was given for that gesture)

For meaningless gestures only:

D (derived) = meaningless action that was created starting from a meaningful one. In brackets is the name of the meaningful action from which it was derived

ND (nonderived) = meaningless gesture created from scratch
