# Supplementary material for: A norming study of high-quality video clips of pantomimes, emblems, and meaningless gestures
Source: Behav Res Methods. 2018 Dec 12;51(6):2817–26. doi: 10.3758/s13428-018-1159-8 (PMC6877486; doi:10.3758/s13428-018-1159-8)
Supplement: Supplementary file 2 — (PDF 69.0 kb) [file 13428_2018_1159_MOESM2_ESM.pdf]

Table 2 Statistical analysis carried out on pantomimes, emblems, and meaningless gestures (American group of raters)

Position	Item's Name	Meaningfulness		Most Frequent Meaning	Meaning Agreement			Alternative Meanings and Percentages of Agreement
		Mean	*Median*		%	*NA*	*H*	
PANTOMIMES (AMERICAN RATERS)								
P1	playing violin	6.98	7	playing violin	100	0	0.00	
P2	driving	6.98	7	driving	100	0	0.00	
P3	calling on a telephone	6.96	7	calling on a telephone	100	0	0.00	
P4	drinking	6.96	7	drinking	100	0	0.00	
P5	smoking a cigarette	6.94	7	smoking	100	0	0.00	
P6	taking pictures	6.91	7	taking a picture	100	0	0.00	
P7	combing 1	6.89	7	combing hair	100	0	0.00	
P8	rocking a baby	6.89	7	rocking a baby	100	0	0.00	
P9	putting on a ring	6.86	7	putting on a ring	100	0	0.00	
P10	pouring from a bottle	6.83	7	pouring	100	0	0.00	
P11	zipping up a jacket	6.83	7	zipping up a jacket	100	0	0.00	
P12	playing guitar	6.80	7	playing guitar	100	0	0.00	
P13	playing golf	6.79	7	playing golf	100	0	0.00	
P14	driving a motorbike	6.77	7	driving/starting a motorcycle	100	0	0.00	
P15	spraying perfume	6.77	7	spraying perfume	100	0	0.00	
P16	turning pages	6.77	7	turning pages while reading	100	0	0.00	
P17	playing basketball	6.76	7	playing basketball	100	0	0.00	
P18	smoking a cigar	6.73	7	smoking	100	0	0.00	
P19	throwing	6.71	7	throwing something	100	0	0.00	
P20	whipping	6.68	7	whipping/stirring	100	0	0.00	
P21	using binoculars	6.62	7	looking through binoculars	100	0	0.00	
P22	changing a lightbulb	6.55	7	changing a lightbulb	100	0	0.00	
P23	using a mobile phone	6.48	7	using a mobile phone	100	0	0.00	
P24	knotting	6.29	7	tying a knot	100	0	0.09	
P25	combing 2	6.36	7	combing hair	98	0	0.11	
P26	putting on earrings	6.76	7	putting on earrings	98	0	0.11	
P27	writing	6.05	6.5	writing/scribbling	98	0	0.12	
P28	shooting with a gun	4.00	5	shooting with a gun	97	0	0.14	
P29	putting on a necklace	6.75	7	putting on a necklace	96	0	0.20	

Table 2 (continued)

Position	Item's Name	Meaningfulness		Most Frequent Meaning	Meaning Agreement			Alternative Meanings and Percentages of Agreement		
		Mean	*Median*		%	*NA*	*H*			
P30	eating with a spoon	6.50	7	eating	96	0	0.20			
P31	filing nails	6.73	7	nail filing	96	0	0.18			
P32	reading	5.52	7	turning pages/reading	93	0	0.27	petting an animal (8)		
P33	dealing cards	6.73	7	dealing cards	91	0	0.30	giving money (9)		
P34	eating with a fork	5.37	7	eating	91	9	0.00			
P35	typing	6.83	7	typing on a keyboard	90	0	0.31	playing piano (10)		
P36	peeling a banana	6.55	7	peeling a banana	90	0	0.38			
P37	putting on glasses	6.49	7	putting on glasses	90	0	0.42	picked up and put a hat on (5)		
P38	seasoning 1	6.34	7	seasoning	89	0	0.38	pouring (14)		
P39	archery	6.64	7	archery	83	0	0.52	using a slingshot (14)		
P40	washing hands	5.13	6.5	washing hands	83	13	0.23			
P41	painting a wall	5.11	6	painting	83	15	0.12			
P42	opening a jar 2	6.71	7	opening a jar	80	0	0.51	screwing on a lid (22)		
P43	spraying deodorant	5.88	6	spraying	79	0	0.71	showing the muscle (12)	pointing the armpit (7)	
P44	sweeping	6.17	7	sweeping	78	0	0.74	rowing (10)	mopping (10)	hoeing (2)
P45	using a corkscrew	6.33	7	removing the cork	77	0	0.76	opening a can/object (12)	pulling apart (9)	
P46	stirring	5.23	7	stirring/mixing	77	19	0.24			
P47	playing drums	5.54	7	paying drums	76	13	0.58			
P48	opening a jar 1	6.67	7	opening a jar	75	25	0.14			
P49	sewing	5.20	6	sewing	74	17	0.40	waving movement (7)		
P50	wiping the mouth	4.56	5	wiping mouth	74	15	0.55			
P51	uncorking a bottle	4.75	5.5	pulling the top off a can/bottle	68	23	0.44			
P52	cutting with a knife	4.75	5	cutting	66	20	0.69			
P53	rowing	4.29	5	rowing a boat	65	24	0.58			
P54	putting on a belt	4.43	5	putting on a belt	61	25	0.76			
P55	applying lipstick	4.34	5	applying lipstick	61	32	0.43			
P56	ironing	4.89	6	ironing	59	20	1.17			
P57	using a remote control	4.72	6	using a remote control	55	26	0.87	giving (11)		
P58	painting nails	4.43	6	painting nails	55	35	0.64			
P59	fastening a security belt	6.02	7	seatbelt	55	0	1.15	putting something in a bag (31)	holster/sheath (7)	
P60	pushing a button	6.38	7	pushing a button	53	0	0.79	pointing at someone (45)		
P61	turning a key	6.19	7	opening a door	52	0	1.28	twisting/screwing something (26)	using a screwdriver (10)	starting a car (10)

Table 2 (continued)

Position	Item's Name	Meaningfulness		Most Frequent Meaning	Meaning Agreement			Alternative Meanings and Percentages of Agreement		
		Mean	*Median*		%	*NA*	*H*			
P62	spraying	4.02	5	spraying	51	36	0.59	scribbling/writing (11)		
P63	playing flute	6.90	7	playing flute	46	0	1.13	playing an instrument (37)	playing a clarinet (12)	
P64	blowing a whistle	5.11	6	blowing a whistle	45	11	1.14	coughing (32)		
P65	blowing nose	4.35	5	blowing nose	45	16	1.27	gross smell (20)	adjusting a mask to snorkel (9)	
P66	cutting with scissors	4.79	5	cutting	45	23	0.78	stapling (30)		
P67	folding fun	4.55	5	spraying perfume	39	27	1.18	using a fan (18)	drying hair (12)	
P68	applying make up	4.91	6	applying make up	39	23	0.80	combing hair (39)		
P69	grinding pepper	6.31	7	grinding/seasoning	38	0	0.98	unscrewing (52)	screwing (7)	
P70	washing hair	4.94	6	washing hair	37	0	1.64	adjusting hair (29)	indicates being nervous/uncomfortable (13)	indicates being confused (8)
P71	playing tennis	4.92	5	playing tennis	37	20	1.39	throwing/tossing a ball (24)	rolling/bowling a ball (8)	
P72	eating an apple	4.13	5	peeling fruit	36	36	1.35	peeling a banana (11)	peeling an orange (9)	turning/flipping (6)
P73	using a hair dryer	4.24	5	hair dryer	33	35	0.92	spraying hairspray/perfume (29)		
P74	erasing**	4.86	5	writing something	58	0	1.51	erasing (11)	sprinkling (8)	
P75	screwing**	5.54	6	turning a key	53	0	0.93	using a screwdriver (40)		
P76	measuring**	4.51	5	zipping	51	22	1.10	measuring (10)	pulling down (10)	
P77	playing cello**	6.66	7	playing violin	34	0	1.40	playing cello (32)	playing a stringed instrument (21)	playing bass (9)
P78	hammering**	4.49	5	knocking	31	20	1.48	hammering (20)	ringing the bell (14)	shaking something (10)
P79	seasoning 2	3.86	4	sprinkling/seasoning	61	35	0.28			
P80	sawing	3.82	4	sawing	45	32	1.31			
P81	wiping	3.79	4	washing (windows)	43	38	1.03	erasing something on a board (6)	turning a wheel (6)	
P82	opening a lighter	3.59	4	using a lighter	39	42	0.93	clicking something (13)		
P83	using a toothpick	3.77	4	using a toothpick	32	34	1.58	picking nose (11)	secret (9)	
P84	lighting a match	3.83	4	lighting a match	19	42	1.40	checkmark (16)	ripping paper (16)	
P85	putting on a watch	3.29	3	putting on a watch	44	45	1.34	putting on a bracelet (33)		
P86	stamping	3.25	2	stamping	25	39	1.41	moving an object from a place to another (23)		
P87	playing cards**	3.72	4	opening and placing	32	28	1.34	opening a wallet and paying (21)	playing cards (13)	
P88	smoking a pipe**	2.21	1	blowing	14	65	1.46	smelling (7)	looking at something closer (7)	
P89	screwing**	1.71	1	wiggling knob	7	75	1.75			
EMBLEMS (AMERICAN RATERS)										
E1	I kill myself	7.00	7	shoot me!	100	0	0.00			

Table 2 (continued)

Position	Item's Name	Meaningfulness		Most Frequent Meaning	Meaning Agreement			Alternative Meanings and Percentages of Agreement
		Mean	*Median*		%	*NA*	*H*	
E2	kiss	7.00	7	blowing a kiss	100	0	0.00	
E3	sleeping	7.00	7	being sleepy/needing sleep	100	0	0.00	
E4	strangling	7.00	7	choking	100	0	0.00	
E5	calling	6.98	7	calling	100	0	0.00	
E6	salute	6.98	7	salute	100	0	0.00	
E7	clapping	6.91	7	clapping	100	0	0.00	
E8	come here	6.91	7	asking someone to come closer	100	0	0.00	
E9	gross smell	6.89	7	indicates a gross smell	100	0	0.00	
E10	pointing	6.87	7	pointing	100	0	0.00	
E11	ok	6.80	7	ok, perfect	100	0	0.00	
E12	counting	6.78	7	counting to five	100	0	0.00	
E13	cutting	6.76	7	cutting/using scissors	100	0	0.00	
E14	walking	6.74	7	walking	100	0	0.00	
E15	disapproving	6.73	7	disapproving	100	0	0.00	
E16	cutting throat	6.71	7	indicates the intention of killing	100	0	0.00	
E17	shouting	6.67	7	shouting/yelling	100	0	0.00	
E18	no	6.66	7	no	100	0	0.00	
E19	four	6.56	7	*four* as a quantity	100	0	0.00	
E20	writing	6.52	7	writing	100	0	0.00	
E21	bowing	6.12	7	bowing	100	0	0.00	
E22	loser	6.55	7	calling someone a loser	98	0	0.11	
E23	finger crossed	6.57	7	finger crossed/ express good luck	98	0	0.11	
E24	approving	6.35	7	approving	98	0	0.11	
E25	looking far away	6.70	7	looking far away	98	0	0.11	
E26	I don't know	6.85	7	I don't know	98	0	0.11	
E27	three	5.90	7	*three* as a quantity	97	0	0.12	
E28	I shoot at you	6.54	7	shooting	94	0	0.23	pointing at you (6)
E29	waving hello 1	6.91	7	saying hi/waving	94	0	0.28	
E30	waving hello 2	7.00	7	waving goodbye	93	0	0.29	indicates lack of interest (4)
E31	stop	6.93	7	stop	93	0	0.29	sending away (4)
E32	speaking	6.38	7	indicates someone that is talking too much	93	0	0.32	

Table 2 (continued)

Position	Item's Name	Meaningfulness		Most Frequent Meaning	Meaning Agreement			Alternative Meanings and Percentages of Agreement			
		Mean	*Median*		%	*NA*	*H*				
E33	money	6.38	7	indicates money	91	0	0.42				
E34	slowing down	5.93	6	slowing down/keeping calm	91	0	0.44				
E35	silence	6.94	7	telling someone to be quiet	90	0	0.38	asking to stop talking (8)			
E36	good guy	4.73	5	patting shoulder to comfort/good job	89	7	0.23				
E37	looking at	6.83	7	looking at you/watching	85	0	0.48	pointing to the eyes (12)			
E38	crazy 3	6.55	7	thinking	79	0	0.83	being clever (10)			
E39	a moment	6.45	7	asking to wait a minute	77	0	0.61	*one* as a quantity (20)			
E40	praying 1	6.26	7	praying	74	0	0.73	namaste (19)	showing gratitude (7)		
E41	so-so	4.52	6	so-so/indicates uncertainty	71	20	0.41	ok (7)			
E42	stomach ache	4.96	5	having a stomach ache	71	0	0.91	bowing (15)	being hungry (7)	being full in the stomach (7)	
E43	being late	6.42	7	indicates that it is late	65	0	0.65	asking "what's the time" (35)			
E44	eating	6.71	7	eating	65	0	0.65	indicates being hungry (35)			
E45	two	6.13	7	*two* as a quantity	62	0	0.84	peace (31)	asking to wait two min (7)		
E46	yawning	6.15	7	yawning	58	0	1.44	covering mouth (13)	sneezing (10)		
E47	listening	6.80	7	listening with more attention	57	0	0.68	indicates that I can't hear you (43)			
E48	exploding1	4.17	5	pulling apart	55	36	0.58				
E49	I care about you	4.32	5	showing comfort/allegiance/care	55	25	0.77	patting shoulder (16)			
E50	getting tired	6.81	7	wiping sweat	52	0	1.08				
E51	triumphing	4.50	5.5	victory/celebration	50	25	0.98	indicates power (16)			
E52	horns	4.04	5	rock on/awesome	49	36	0.95				
E53	praying 2	4.20	5	praying	47	35	0.86	holding hands (10)	indicates togetherness (6)		
E54	idea	4.57	5	getting an idea	45	23	1.45	indicates thinking (11)			
E55	hitchhiking	6.10	7	hitchhiking	44	0	1.20	"get out" (32)	pointing left or right (10)	indicates a person on the right/left (7)	drawing attention to something to her side (7)
E56	sated	6.26	7	being full in the stomach	31	0	1.23	bring hungry (49)	indicates that the food was delicious (16)		
E57	smelling	4.22	5	smelling	31	35	1.43	touching the nose (18)	indicates knowing something (6)		
E58	snapping fingers	4.21	5	snapping fingers	19	36	1.92	performed when something is done quickly (17)	indicating disappointment (6)	catching the attention (6)	
E59	anger**	6.56	7	yawning	72	0	0.81	indicates being nervous/scared (21)			
E60	quietly**	6.06	7	stop	74	0	0.85	honk (15)	pushing someone away (7)		

Table 2 (continued)

Position	Item's Name	Meaningfulness		Most Frequent Meaning	Meaning Agreement			Alternative Meanings and Percentages of Agreement			
		Mean	*Median*		%	*NA*	*H*				
E61	winning**	6.67	7	peace signal	67	0	0.84	*two* as a quantity (27)			
E62	forgetting**	6.66	7	"stupid me"	64	0	0.93	facepalm (24)	indicates that something was obvious (9)		
E63	begging**	5.72	6	giving/offering	84	0	0.73	showing (9)	getting (5)		
E64	rub one's hands**	5.16	6	washing hands	82	14	0.22				
E65	I don't know anything**	4.87	6	its wasn't me	28	0	1.75	I don't know (23)	defensive manner (23)	stopping (10)	surrendering (8)
E66	agreement**	4.96	6	"I see you, I got my eyes on your"	26	21	1.57	crying/tears (23)	looking (15)	pointing eyes (9)	
E67	standing up**	4.39	5	lifting up/rising	76	0	0.78	asking more or higher amount (14)	standing up (8)		
E68	closer**	4.04	5	kicking away	57	36	0.43				
E69	congratulations**	4.67	5	shaking hands	48	15	1.24	having an agreement (20)	indicates friendship (9)		
E70	drinking	3.94	4	drinking	51	39	0.61	drink alcohol (6)			
E71	union	3.63	4	bonding together	28	47	1.40	joining (11)			
E72	lowering	3.43	3	lowering down	55	39	0.41				
E73	growing up	3.32	3	rising up	32	41	1.19	growing up (16)			
E74	exploding 2	2.96	3	explosion	30	43	1.31	disappear (10)	pull apart (7)	moving hands apart (7)	
E75	tasty	3.10	3	smiling	20	41	1.78	cute (18)	showing dimples (6)		
E76	sending away	3.22	1.5	move away	26	53	1.12	cleaning/sweeping (15)			
E77	inverting	2.69	1	flipping over	39	55	0.55				
E78	thinking	2.46	1	thinking	30	53	0.88				
E79	dying**	3.86	4	cross sign	41	35	1.17	cutting (12)			
E80	caress**	3.55	4	wiping face	29	41	1.63				
E81	putting together**	3.59	3.5	pinch	30	33	1.18	small amount (27)			
E82	crazy 1**	3.35	3	thinking	31	41	1.12	saying that someone is crazy (18)	indicates being smart (6)		
E83	I beat you**	2.15	1	cutting/slicing	25	69	0.72				
E84	cunning**	1.87	1	crying/being sad	20	74	0.72				
E85	later**	2.94	1	spinning/roll over	19	53	1.64	continue (13)			
E86	stealing**	1.83	1	grab/grasp	17	74	0.98				
E87	swearing**	2.74	1	stay quiet	17	64	1.05	shut up (15)			
E88	run away**	2.57	1	shaking hands	16	65	1.47	splitting (6)	go away (6)		
E89	coupling**	1.77	1	connecting two things together	15	72	1.41				
E90	skinny**	1.63	1	showing one finger	15	75	1.23				
E91	to bear a grudge**	3.02	1	wrap up/hurry up	14	55	1.70	winding (14)	spinning motion (6)		
E92	getting crazy**	1.55	1	turn/twist	14	80	1.00				

Table 2 (continued)

Position	Item's Name	Meaningfulness		Most Frequent Meaning	Meaning Agreement			Alternative Meanings and Percentages of Agreement
		Mean	*Median*		%	*NA*	*H*	
E93	finished**	1.25	1	twisting/screwing	10	80	0.97	gun (7)
E94	collision**	1.20	1	connecting together	9	80	1.58	
E95	fear**	2.13	1	pinching	9	68	1.97	
E96	long time ago	1.33	1	putting a situation behind	7	85	1.04	
E97	I don't care**	1.62	1	telling someone off	7	76	1.55	related to chin (7)
E98	being alone**	2.70	1	indicate money	7	52	2.66	
E99	arresting**	1.84	1	crossing arms	7	76	1.85	
E100	crazy 2**	1.57	1	waving hands	5	84	1.55	
E101	hungry**	1.02	1	hand movement	4	96	0.00	
E102	what do you want?**	1.93	1	give me	3	83	1.61	
E103	full of people**	1.05	1	give me money	2	98	0.00	
E104	unpleasant**	1.14	1	hand down	2	93	1.10	
MEANINGLESS (AMERICAN RATERS)								
M1	ND_07	1.00	1		0	100		
M2	D_05 (peeling a banana)	1.07	1			90		
M3	ND_14	1.09	1			91		
M4	ND_18	1.11	1			96		
M5	ND_20	1.12	1			95		
M6	ND_21	1.14	1			93		
M7	ND_29	1.14	1			84		
M8	ND_30	1.14	1			95		
M9	ND_13	1.15	1	indicates togetherness	4	89	1.33	
M10	ND_01	1.17	1	Showing a ring	6	87	1.04	
M11	ND_09	1.18	1	indicates that a person is two faced	6	79	1.75	
M12	ND_37	1.19	1	hand under the chin	7	88	0.95	
M13	D_28 (using a hair dryer)	1.21	1	combing the hair	7	88	0.95	
M14	ND_06	1.21	1	headache	11	82	0.95	
M15	D_16 (sweeping)	1.22	1			91		
M16	ND_35	1.24	1	salute	8	89	0.91	
M17	D_18 (writing 1)	1.25	1			91		
M18	ND_23	1.28	1			93		
M19	D_02 (playing flute)	1.30	1	playing an instrument	23	67	0.94	

Table 2 (continued)

Position	Item's Name	Meaningfulness		Most Frequent Meaning	Meaning Agreement			Alternative Meanings and Percentages of Agreement	
		Mean	*Median*		%	*NA*	*H*		
M20	D_33 (screwing)	1.31	1			87			
M21	ND_02	1.31	1	animal horns—antler	15	94	1.25	saying hello (6)	finger head (6)
M22	ND_15	1.32	1			86			
M23	D_25 (using binoculars)	1.34	1	lifting something up	7	86	1.01		
M24	D_04 (cutting with a knife)	1.37	1	using a lasso	10	58	1.95	making a loop (6)	
M25	ND_26	1.39	1			88			
M26	ND_08	1.40	1	showing front and back	17	67	1.36	indicates to stop at this point (6)	flipping over something (6)
M27	ND_17	1.41	1	indicates sadness/crying	11	83	0.90		
M28	D_32 (sewing)	1.43	1			78			
M29	ND_03	1.51	1	drawing a spiral shape	35	53	0.91		
M30	D_10 (praying 1)	1.52	1	being together	18	70	1.23		
M31	ND_32	1.52	1	fist over fist	14	69	1.20	stacked/locked (10)	
M32	ND_25	1.60	1	expressing good luck	14	81	0.74		
M33	D_21 (calling on a telephone)	1.61	1	ringing a chime	7	80	1.31	spinning motion (7)	
M34	ND_28	1.61	1	indicating the level	9	82	1.39		
M35	ND_11	1.63	1	ok	24	67	0.89		
M36	D_13 (driving)	1.65	1	turning a valve overhead	24	72	0.54		
M37	D_22 (combing 2)	1.67	1	spraying	7	72	2.10		
M38	D_27 (praying 2)	1.67	1	pressing the forehead	7	69	1.95	indicates togetherness (7)	
M39	D_09 (painting nails)	1.71	1	writing down something	20	63	1.26	painting nails (9)	
M40	D_11 (opening a lighter)	1.71	1	taking a picture	19	65	0.92	spraying (13)	
M41	D_08 (sleeping)	1.74	1	being sleepy	35	46	1.16	tilting the head (8)	
M42	ND_19	1.76	1	yes or no—maybe	13	74	1.13	thumbs in and out (9)	
M43	D_19 (waving hello)	1.79	1	"Can't see me "- John Cena	9	74	1.47	waving (7)	
M44	D_30 (writing 2)	1.84	1	scratching	16	70	1.41		
M45	D_23 (cleaning)	1.89	1	wiping/cleaning	14	70	1.63		
M46	D_07 (turning a key)	1.93	1	turning/twisting something	32	44	1.09	snapping fingers (12)	unscrewing (9)
M47	ND_36	1.93	1	ok	13	68	1.84		
M48	D_15 (applying lipstick)	2.07	1	applying make-up/cream	26	65	0.70	drawing something on the face (7)	
M49	D_37 (listening)	2.07	1	listening	20	70	0.94		
M50	D_06 (wearing glasses)	2.14	1	putting on a mask	43	37	0.62	putting on a party hat (17)	
M51	ND_33	2.17	1	grabbing	24	62	0.66	closing fist (12)	

Table 2 (continued)

Position	Item's Name	Meaningfulness		Most Frequent Meaning	Meaning Agreement			Alternative Meanings and Percentages of Agreement			
		Mean	*Median*		%	*NA*	*H*				
M52	ND_22	2.18	1	indicating the direction	7	80	2.50				
M53	ND_24	2.22	1	waving someone away	20	64	1.12	dismissing (7)	waving (7)		
M54	D_12 (grinding pepper)	2.29	1	screwing	13	60	1.48	grinding pepper (11)	unscrewing (7)	opening jar (7)	
M55	D_26 (walking)	2.33	1	walking/running	26	56	1.19	flying (9)			
M56	D_01 (eating with a spoon)	2.37	1	scooping	21	24	1.82	pouring something (15)	putting on a hat (12)	washing hair (9)	bathing (6)
M57	D_20 (brushing teeth)	2.41	1	pounding	9	61	2.12	brushing teeth (7)	cutting (7)		
M58	D_03 (combing 1)	2.50	1	brushing with a lint roller	55	34	0.44	shaving arms (10)			
M59	ND_27	2.72	1	telling a secret	15	62	1.57	don't look (9)	resting/sleeping (6)		
M60	D_31 (pouring from a bottle)	2.75	1	flipping over	20	55	1.44	pouring out something (11)	opening jar (7)		
M61	ND_16	2.76	1	making fun	16	61	1.18	drinking (14)	elephant symbol (6)		
M62	D_17 (winning)	2.78	1	pointing at you	39	57	0.31				
M63	ND_04	2.80	1.5	coming together/joining	38	33	1.08	indicates that something is interlocked together (13)			
M64	D_24 (erasing)	2.98	1	cleaning/wiping	30	57	0.61	smoothing (13)			
M65	ND_34	3.23	2.5	flipping something over	45	64	0.94				
M66	D_14 (smoking)	3.27	1	smoking	41	53	0.47				
M67	ND_12	3.47	4	covering eyes not to see	35	35	0.93	shielding eyes (24)	fainting (6)		
M68	ND_31	3.52	4	togetherness	45	34	0.95	locking hands (9)			
M69	ND_40	3.57	4	checking fever	41	39	1.09	headache (7)			
M70	ND_5	3.61	3.5	stop	52	48	0.00				
M71	ND_10	3.65	3.5	touching the nose	26	42	1.40	be quiet (13)	correct (10)	drunk test (6)	
M72	D_36 (washing hair)	3.93	4	rubbing hurt shoulder	45	25	1.19	rubbing/scratching shoulder (11)	consoling/it's ok (11)		
M73	D_35 (stirring)	4.09	4.5	twirling something	32	27	1.48	lasso (20)	celebrating (9)		
M74	D_34 (triumphing)	4.14	5	threatening for a fight	22	31	1.77	anger (16)	making a fist (12)	be strong (6)	
M75	ND_38	4.28	4.5	removing the ring	58	29	0.58	take off something (10)			
M76	D_29 (calling)	4.43	6	hang loose	34	39	1.46	call me (9)	cool (7)		
M77	ND_39	4.73	5	waving/water motion	37	22	1.06	swimming motion (33)			
